# Who prefers what? Correlates of preferences for next‐generation HIV prevention products among a national U.S. sample of young men who have sex with men

**DOI:** 10.1002/jia2.26096

**Published:** 2023-07-13

**Authors:** Katie B. Biello, Pablo K. Valente, Daniel Teixeira da Silva, Willey Lin, Ryan Drab, Lisa Hightow‐Weidman, Kenneth H. Mayer, José A. Bauermeister, the iTech Team

**Affiliations:** ^1^ Departments of Behavioral & Social Sciences and Epidemiology, School of Public Health Brown University Providence Rhode Island USA; ^2^ Center for Health Promotion and Health Equity Brown University Providence Rhode Island USA; ^3^ The Fenway Institute Fenway Health Boston Massachusetts USA; ^4^ Department of Allied Health Sciences University of Connecticut Waterbury Connecticut USA; ^5^ Department of Family & Community Health University of Pennsylvania School of Nursing Philadelphia Pennsylvania USA; ^6^ College of Nursing Florida State University Tallahassee Florida USA

**Keywords:** men who have sex with men, adolescents, HIV prevention, pre‐exposure prophylaxis, conjoint experiment, product preferences

## Abstract

**Introduction:**

Pre‐exposure prophylaxis (PrEP) has been available for young people for over a decade, yet only ∼15% of young people in the United States with indications for PrEP have a prescription for it. Next‐generation PrEP modalities may address some of the challenges of daily oral PrEP. However, preferences for these products are unknown.

**Methods:**

From October 2020 to June 2021, we conducted an online survey of 737 cisgender, young men who have sex with men (age 15–24 years) without HIV across the United States who reported same‐sex attraction or consensual sex with another man in the past 6 months. Participants completed a conjoint experiment comparing daily oral pills, event‐driven oral pills, event‐driven rectal douches, intramuscular injections, intravenous broadly neutralizing antibody (bnAb) infusions and subcutaneous implants. Participants ranked the products from most to least preferred. Exploded logit models examined the association between ranked preferences of PrEP modalities and socio‐demographic and behavioural characteristics.

**Results:**

Participants’ mean age was 21 years (SD = 2.3), and 56% identified as White. Nineteen percent were currently taking daily oral PrEP, and another 9% had previously taken it. Participants prioritized efficacy, absence of side effects and costs in the conjoint analyses. Daily oral PrEP had the highest preference ranking, followed by event‐driven oral (OR = 0.89, *p* = 0.058), injectable (OR = 0.83, *p* = 0.005), implant (OR = 0.48, *p* < 0.0001), bnAb infusions (OR = 0.38, *p* < 0.0001) and rectal douches (OR = 0.24, *p* < 0.0001). There were differences in PrEP preferences across age, insurance status, sexual behaviour, PrEP use history, HIV and sexually transmitted infection (STI) testing history, and STI diagnoses (omnibus tests: *p* < 0.05). Participants also provided reasons for selecting their top product choice: ease of use for those who chose daily oral (99%) and daily event‐driven (98.5%); feel more protected against HIV for those who chose injectable (95.4%) and implants (100%); not worrying about forgetting to take it for those who chose bnAbs (93.8%); and being able to stop taking it when they want for those who chose rectal douche (90.9%).

**Conclusions:**

Next‐generation modalities were less likely to be preferred over daily oral PrEP, with differences in the magnitude by socio‐demographic and behavioural characteristics. Given the low uptake of daily oral PrEP, end‐users’ preferences for and concerns about PrEP products must be understood to ensure high acceptability and penetration.

## 1 INTRODUCTION

HIV incidence continues to be a major public health concern among young men who have sex with men (YMSM) in the United States, who account for 24% of new infections among MSM [1]. In May 2018, the antiretroviral combination tenofovir‐emtricitabine, prescribed as a daily oral pill, was approved by the U.S. Food and Drug Administration (FDA) for HIV pre‐exposure prophylaxis (PrEP) for adolescents weighing at least 77 lb (35 kg), almost 6 years after approval for adults [2, 3]. With greater than 90% efficacy when taken as prescribed, PrEP is an essential tool in ending the HIV epidemic [4, 5]. However, PrEP uptake has been low among adolescents and young adults [6, 7, 8, 9]. Only 2% of individuals 13–26 years old meeting indications for PrEP have been prescribed the medication in the United States [10], with adolescents under 18 years old accounting for less than 1% of all PrEP prescriptions [11].

Moreover, a recent meta‐analysis showed that, across studies, about one‐third of YMSM who initiated PrEP have suboptimal adherence [6, 7, 12]. Barriers to PrEP uptake and adherence among YMSM are multi‐level and include individual (e.g. forgetting/hectic lifestyles and concerns about side effects) [13, 14, 15, 16, 17], interpersonal (e.g. stigma and discrimination from family, friends and sexual partners) [13, 17, 18] and structural (e.g. access to health insurance and concerns about cost) factors [13, 15, 19, 20].

New PrEP formulations, including implants and long‐acting injectable products, may help address challenges individuals face adhering to a daily oral pill [21, 22, 23, 24]. Diverse drug delivery mechanisms could offer choices based on YMSM's socio‐cultural context and facilitate uptake of and adherence to products that are behaviourally congruent with their sexual practices. For example, rectal douches may be congruent with cleansing practices and behaviours regarded as normative before participating in activities where the probability of engaging in receptive anal intercourse is high [25, 26]. Similarly, long‐acting injectable formulations or episodic oral PrEP might be more compatible with a complex lifestyle when taking a pill daily is unrealistic.

Achieving consistent and correct use among the product's consumers will require researchers to develop desirable and acceptable products to end‐users within the context of clinical trials and in the real world [27, 28, 29]. However, YMSM's recruitment in these next‐generation PrEP studies has been limited [3], and little is known about how YMSM perceive next‐generation prevention modalities or the factors that influence the acceptability of these products in this population. Thus, even if found to be efficacious, the absence of YMSM's perspectives on product characteristics could lead to acceptability and adherence challenges once they become available as HIV prevention strategies.

We conducted an online survey with U.S. YMSM examining PrEP modality preferences to address this gap. In this study, we aim to examine the (1) preferences for features of PrEP products *within* YMSM participants via conjoint analyses and (2) across PrEP product preferences and correlates of these preferences *between* YMSM participants (via rank‐choice selection).

## 2 METHODS

### 2.1 Participants and procedures

YMSM in the United States were recruited for an online survey from social media platforms, dating apps and targeted mailing lists between October 2020 and June 2021. Participants were eligible for this study if they were 15–24 years old, were assigned male sex at birth and currently identified as male, self‐reported being HIV negative or did not know their HIV status, reported same‐sex attractions or consensual sexual behaviours with other men in the past 6 months, lived in the United States and spoke English.

Interested individuals completed an online screener to ascertain eligibility. We used automatized (e.g. CAPTCHA validation) and manual methods (e.g. time to complete screener) to verify the legitimacy of responses and checked phone numbers and email addresses to identify duplicate entries, as recommended by previous research [30, 31, 32]. Eligible individuals then received a unique, password‐protected link to an online survey developed and pilot‐tested with a Youth Advisory Board of sexual and gender minority youth. Individuals were randomized to a brief conjoint experiment exploring preferred attributes of one of five different next‐generation modalities (i.e. event‐driven oral, intramuscular injections, subcutaneous implants, intravenous infusions of broadly neutralizing antibodies [bnAbs] and rectal douches). Conjoint experiments can shed light on HIV prevention decision‐making by examining how product attributes (e.g. efficacy and side effects) influence choice preferences [33]. Following the conjoint experiment, the survey assessed ranked preferences across daily oral and next‐generation PrEP modalities, and socio‐demographic and behavioural characteristics. The survey was designed to be completed in ≈45 minutes.

The Institutional Review Board at the University of Pennsylvania approved study procedures and materials. Informed consent/assent was obtained from all participants, and a waiver of parental consent was obtained for participants 15–17 years old. Participants received a USD$40 gift card for participation.

### 2.2 Measures

#### 2.2.1 Conjoint experiment

**Figure 1:**
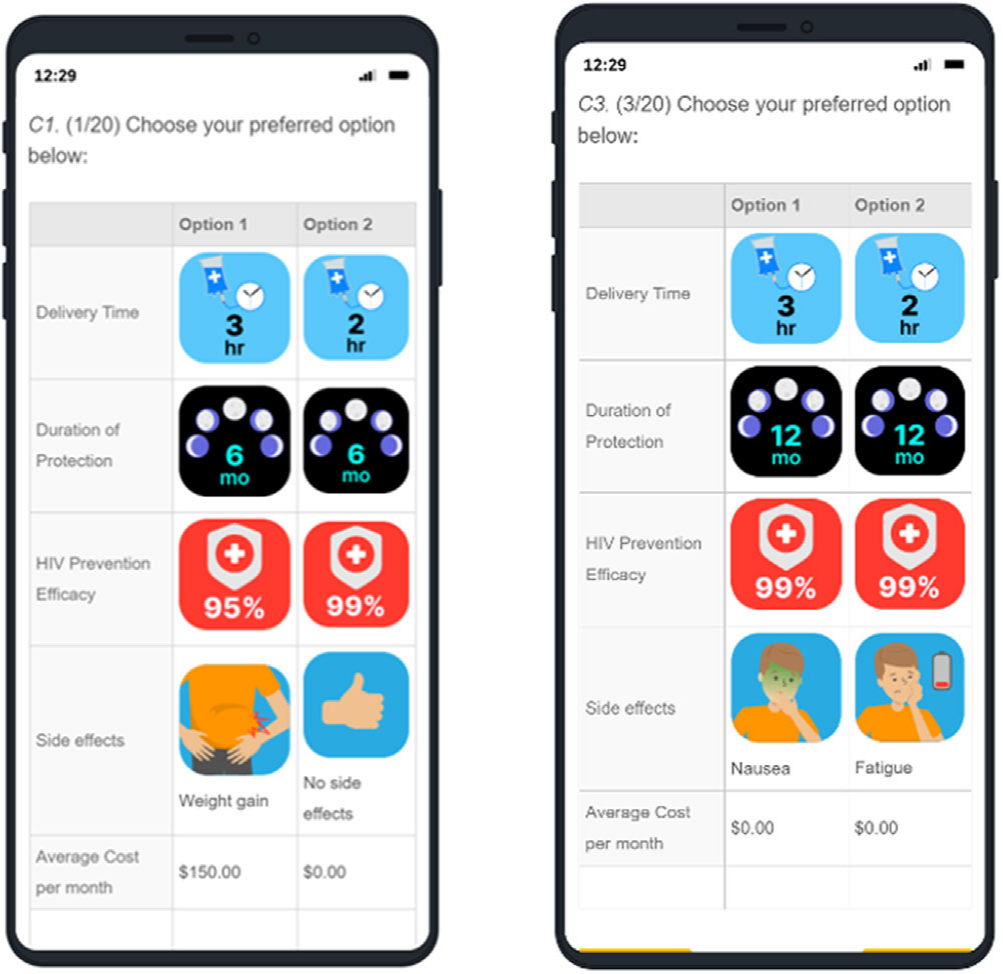
Sample of two product profiles from the conjoint experiment. Abbreviations: hr, hour; mo, month.

Definitions of PrEP products and images for the product features were reviewed by participants prior to the conjoint experiment. Definitions and images were developed through cognitive interviews with YMSM (reported previously [34]). Given that many of the products are still under development, and that we were trying to assess optimal attributes, we did not include the frequency of recommended use in the descriptions (e.g. bimonthly for injectable PrEP). Participants then selected between 20 pairs of product profiles based on random combinations across five or six product‐specific features. All conjoint experiments assessed HIV prevention efficacy, duration of protection, average cost and side effects. Additionally, event‐driven oral PrEP experiments assessed pill size and timing of use; injectable experiments assessed deliverer; implant experiments assessed time for placement; bnAb experiments assessed infusion time; and rectal douche experiments assessed delivery device. Figure 1 shows a sample of a conjoint experiment.

#### 2.2.2 PrEP modality preferences

Following the conjoint experiment, participants were then asked to rank each modality (including daily oral PrEP) from best (1) to worst (6) if they provided equal protection against HIV transmission based on their preferences and how the product would fit their lifestyles.

We then asked participants to indicate on a 4‐point scale their agreement with 11 reasons they selected their top‐ranked PrEP modality. Sample reasons included, “It would be easy to use this product,” “I would have fewer concerns about side effects,” and “I feel more protected against HIV using this product.” We further dichotomized responses to each statement as agree/strongly agree versus disagree/strongly disagree.

#### 2.2.3 Access to healthcare

We classified health insurance status as uninsured, covered by their parents’/guardians’ insurance plan and covered by their own insurance plan. We assessed whether participants had a primary care provider and the extent to which participants believed the healthcare services accessed met their health needs. Responses on a 5‐point scale ranging from “never” to “always” were dichotomized as “usually/always” and “never/rarely/sometimes” meet health needs.

#### 2.2.4 Access to HIV prevention care

We assessed previous HIV and other STI testing, prior diagnosis of STIs, awareness of PrEP for HIV prevention and PrEP use (current use, prior use or never used).

#### 2.2.5 Sexual and drug use behaviours

Participants were asked about the number of men with whom they had condomless anal sex (CAS) in the past month, with responses dichotomized into any versus none. Participants were also asked about any use of the following non‐prescribed substances in the past 30 days: cocaine/crack cocaine, stimulants, inhalants, sedatives, hallucinogens and opioids. Our composite measure of drug use included any use of these substances in the past 30 days. Lastly, participants were asked how frequently they had a drink containing alcohol in the past 30 days, which was dichotomized into 2+ drinks per week versus less than 2 drinks per week.

#### 2.2.6 Socio‐demographics

We also assessed participants’ age, race, ethnicity, sexual orientation, educational attainment and ZIP code (coded into Census regions). To measure food insecurity, we asked participants how often they or their families had to skip a meal in the past 3 months because there was not enough money for food. Responses on a 4‐point scale ranging from “almost every week” to “never” were dichotomized into any versus no food insecurity.

### 2.3 Analytic plan

We described frequencies and descriptive statistics for socio‐demographic and behavioural characteristics.

Using Qualtrics Conjoint Experiment platform, we estimated the feature importance of each attribute in the participants’ decision‐making process. We also calculated YMSM's willingness‐to‐pay (WTP; i.e. average cost per month in $USD) for a feature based on trade‐offs between different levels.

We described ranking frequencies and mean ranking across all six PrEP modalities and frequencies of endorsement of reasons for selecting the top‐ranked PrEP modality. We used rank‐ordered regression (e.g. exploded logit regression) to model the probability of ranking each modality highest [35], which we present alongside participant‐reported top‐ranked modality as they provide complementary information. Specifically, participant‐reported top‐ranked choices describes the one modality participants would choose if all options were accessible to them. In contrast, model‐estimated probabilities account for the distribution of ranks across products, including bimodal ranking (e.g. a high percentage of both acceptance and rejection).

Rank‐ordered regression is a generalization of conditional logit models that estimates the preference for a given PrEP modality compared to an arbitrarily defined reference category based on sets of ranked items. Following the procedures outlined by Allison and Christakis [36], we used partial likelihood procedures for proportional hazards to estimate rank‐ordered models and examine heterogeneity in PrEP modality preferences across participant characteristics (e.g. if participants’ socio‐demographic characteristics are associated with modality preferences). In these models, variables of interest were included in regression models as interaction terms “variable*modality,” and significance of associations was assessed by Wald chi‐square tests of the hypothesis that all variable*modality terms are zero. Exponentiated regression coefficients of rank‐ordered models are interpreted as odds ratios (ORs) of preferring a given next‐generation modality over daily oral PrEP. Likewise, exponentiated differences in coefficients are interpreted as the odds of preferring a given modality over daily oral PrEP between levels of the variable of interest [36].

We used an exploratory approach to model building, first examining bivariate associations between modality preferences and several measures of socio‐demographic characteristics, access to healthcare and HIV prevention, and sexual and drug use behaviours selected based on our previous research on acceptability and access to PrEP. We then retained variables with *p*‐values <0.10 in bivariate analysis for adjusted models. STI diagnosis and alcohol use were both excluded from the adjusted models due to high collinearity with HIV testing and drug use, respectively. Given that ORs in adjusted rank‐ordered models assume reference levels of other covariates in regression models and that ORs are non‐collapsible [37], the magnitudes of adjusted and unadjusted ORs are not comparable. We, therefore, present unadjusted ORs and report only *p*‐values of adjusted ORs.

Analyses were done in SAS 9.4 and Stata 17.

## 3 RESULTS

Table 1 shows sample characteristics. In brief, the mean age of our sample of *N* = 737 YMSM was 21.1 years (Standard Deviation = 2.27). Nearly, one‐quarter (24%) identified as Latino. Over half (56%) identified as White, 19% as Black, 12% as Asian and 13% as Multiracial or some other racial identity. While nearly all (95%) were aware of PrEP, only 28% had ever used PrEP.

**Table 1:** Characteristics of an online sample of young men who have sex with men in the United States (*N* = 737)

	*N* (%)
Age (years)	
15–17 years	61 (8%)
18–20 years	200 (27%)
21–24 years	475 (65%)
Race	
White	412 (56%)
Black	139 (19%)
Asian	91 (12%)
Multiracial/Other	95 (13%)
Latinx/Hispanic	174 (24%)
Sexual orientation	
Gay	587 (80%)
Bisexual	111 (15%)
Queer	29 (4%)
Other	9 (1%)
Region	
South	251 (34%)
Northeast	209 (29%)
West	137 (19%)
Midwest	134 (18%)
Education attainment	
6–11 grade	67 (9%)
Graduated high school/GED	107 (15%)
Some college/technical school	250 (34%)
Graduated from 2‐ or 4‐year college or technical school	224 (31%)
Some graduate school or higher	87 (12%)
Food insecurity (past 3 months)	134 (18%)
Insurance status	
Parents’ insurance	450 (61%)
Own insurance	219 (30%)
Uninsured	57 (8%)
Don't know	9 (1%)
Has healthcare provider	591 (84%)
Healthcare meets health needs	568 (81%)
HIV test	
Ever	582 (79%)
Past 3 months	294 (42%)
STI test	
Ever	497 (71%)
Past 3 months	252 (36%)
STI diagnosis (ever)	172 (25%)
PrEP awareness	698 (95%)
PrEP use	
Current use	130 (19%)
Past use (not current)	66 (9%)
Condomless anal sex (past 6 months)	315 (45%)
Drug use (past month)	95 (14%)
Alcohol use, 2+ days per week (past month)	168 (24%)

Abbreviations: GED, General Education Development Test; HIV, human immunodeficiency virus; PrEP, pre‐exposure prophylaxis; STI, sexually transmitted infection.

### 3.1 Preferred attributes: a series of conjoint experiments

See Table 2 for the results of the conjoint experiments. Across all PrEP products presented in the conjoint experiment, the most important features were: efficacy (ranging from 33% to 42%), side effects (ranging from 18% to 33%) and average cost (ranging from 18% to 20%); however, side effects had a higher proportion for event‐driven products (e.g. event‐driven oral and rectal douche; 30% and 33%) compared to systemic products (e.g. injectable, implants and bnAb; 18%–23%). Duration of protection had greater feature importance for non‐event‐driven products (14%–18%) than for event‐driven products (4% and 6%). Timing of use (e.g. around sex) or timing of delivery (e.g. length of infusion) demonstrated low feature importance (<3%) across all products, as did the mode of delivery (2%–6%).

**Table 2:** Feature preference for next‐generation PrEP products among young men who have sex with men in the United States

	Douche (*n* = 139)			On‐demand oral PrEP (*n* = 145)			Injectable PrEP (*n* = 139)			PrEP implant (*n* = 146)			bnABs (*n* = 144)		
Feature		Feature importance (%)	WTP (in $USD)		Feature importance (%)	WTP (in $USD)		Feature importance (%)	WTP (in $USD)		Feature importance (%)	WTP (in $USD)		Feature importance (%)	WTP (in $USD)
Average cost per month		20.2			18.8			20.3			17.8			20.00	
Delivery		6.3			2.1			3.1			3.6				
	Hose		−13.70	0.5 inch pill		Ref.	Self‐injected		−12.20	Self‐inserted		−19.00			
	Enema		Ref.	1 inch pill		−12.50	Provider		Ref.	Provider		Ref.			
	Bulb		6.40												
Duration		4.3			6.1			17.7			13.7			17.9	
	6–24 hours		Ref.	6–24 hours		Ref.	1 month		−37.30	1 month		Ref.	1 month		Ref.
	1–2 days		6.00	1–2 days		11.00	2 months		Ref.	2 months		33.80	2 months		38.70
	3–5 days		25.80	3–5 days		24.20	4 months		57.20	4 months		50.20	3 months		66.70
	6–7 days		32.20	6–7 days		33.50	6 months		95.80	6 months		84.00	6 months		96.10
							12 months		122.70	12 months		114.20	12 months		131.60
Efficacy		33.3			40.4			39.0			42.1			40.2	
	50%		−225.70	50%		−267.10	50%		−250.80	50%		−298.00	50%		−259.20
	65%		−185.00	65%		−185.90	65%		−181.80	65%		−212.50	65%		−181.90
	80%		−59.60	80%		−84.50	80%		−80.10	80%		−88.60	80%		−81.40
	95%		Ref.	95%		Ref.	95%		Ref.	95%		Ref.	95%		Ref.
	99%		49.70	99%		54.90	99%		39.90	99%		53.90	99%		45.20
Side effects		33.4			30.4			20.00			22.9			18.5	
	None		Ref.	None		Ref.	None		Ref.	None		Ref.	None		Ref.
	Nausea		−119.50	Nausea		−91.50	Nausea		−54.10	Nausea		−97.40	Nausea		−56.00
	Diarrhoea		−123.40	Diarrhoea		−114.80	Fever		−46.40	Fever		−70.70	Fever		−45.00
	Fatigue		−67.90	Fatigue		−50.80	Fatigue		−41.30	Fatigue		−60.20	Fatigue		−29.00
	Kidney decline		−261.70	Kidney decline		−214.50	Soreness at injection site		−27.50	Soreness at insertion site		−28.80	Soreness at injection site		−35.10
	Weight gain		−154.80	Weight gain		−123.80	Weight gain		−132.50	Weight gain		−175.60	Weight gain		−123.90
Timing of use		2.6			2.2										
	30 minutes before sex		Ref.	2–24 hours before sex + for 2 days after sex		Ref.									
	30–60 minutes before sex		1.70	1–2 hours before sex + for 2 days after sex		9.00									
	1–2 hours before sex		1.70	2–24 hours before sex + for 1 day after sex		11.40									
	2–4 hours before sex		−12.50	1–2 hours before sex + for 1 day after sex		16.60									
Timing for delivery														3.4	
													30 minutes		Ref.
													1 hour		−2.50
													2 hours		−9.10
													3 hours		−25.30

Abbreviations: bnABs, broadly neutralizing antibodies; PrEP, pre‐exposure prophylaxis; USD, United States Dollar; WTP, willingness to pay.

The willingness to pay metric indicates that efficacy and side effects had the greatest cost trade‐offs in participants’ choices. YMSM were less willing to adopt a given technology in the presence of lower efficacy and certain side effects, whereas they were more forgiving of the mode of delivery, duration of HIV protection and timing of regimen use. For example, across products, participants were willing to pay >$250 more for a product with 95% efficacy compared to 50% efficacy. Additionally, a negative impact on kidney function was seen as a major trade‐off for the event‐driven products (>$200 compared to no side effects), as was weight gain for all products ($125–$176).

### 3.2 Preferred products: rank orders

**Figure 2:**
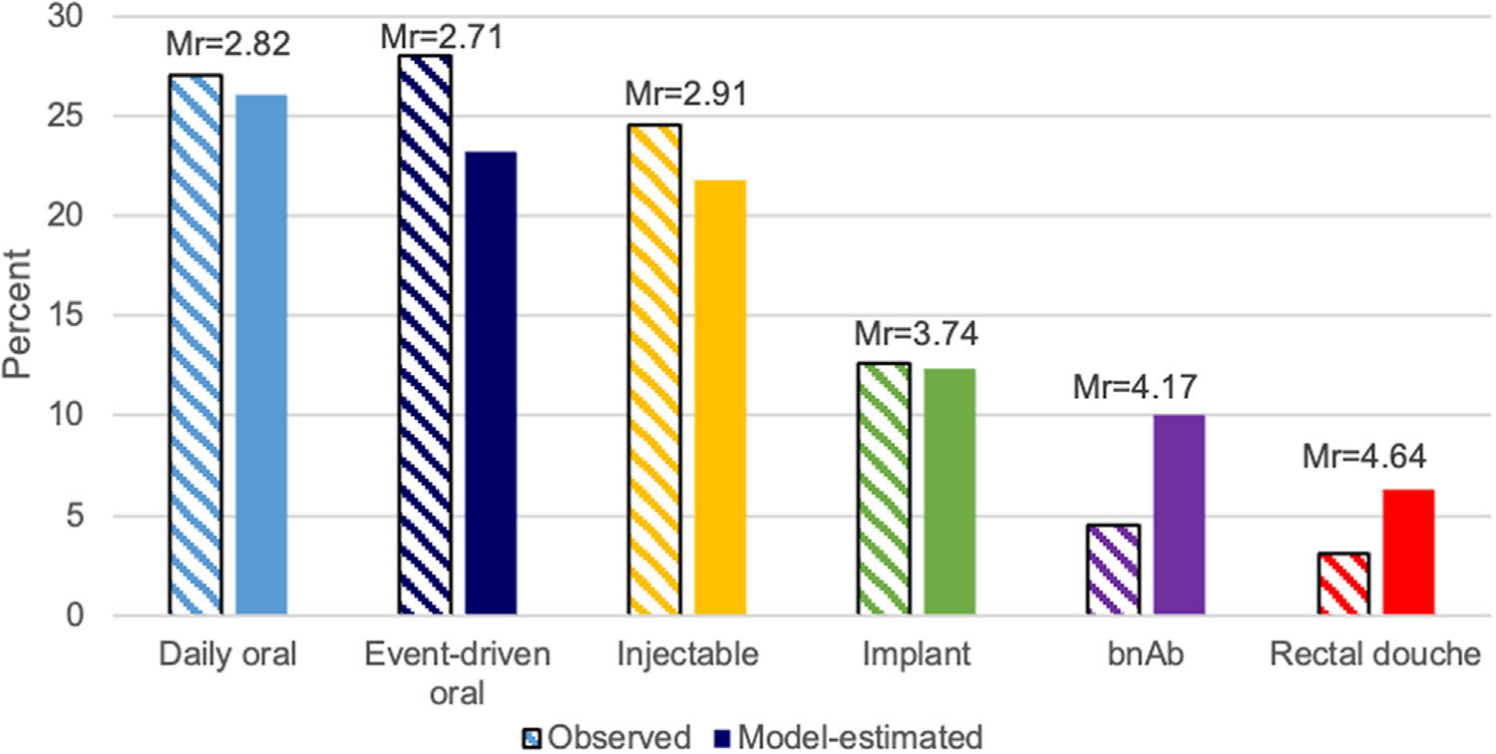
Observed and model‐estimated top‐ranked preferences of PrEP modalities among young men who have sex with men in the United States. Abbreviations: bnABs, broadly neutralizing antibodies; Mr, mean ranking.

The complete ranks by each product are described in Table 3. Approximately one‐quarter of participants ranked event‐driven oral PrEP (28%), daily oral PrEP (27%) and injectable PrEP (24.6%) as their first choice of products, followed by PrEP implant (13%), bnAb infusion (4%) and a PrEP rectal douche (3%). When accounting for the complete rank order of the products (i.e. ranks 1–6), the model‐estimated proportion of individuals selecting daily oral PrEP was 26%, 23% for event‐driven oral PrEP, 22% for injectable PrEP, 12% for PrEP implant, 10% for bnAb infusion and 6% for PrEP rectal douche. Figure 2 shows the observed and model‐estimated top‐ranked rankings of PrEP modalities.

**Table 3:** Next‐generation PrEP product preference rankings among young men who have sex with men in the United States

	Ranking					
Product	1st	2nd	3rd	4th	5th	6th
Daily oral PrEP	27.1%	23.4%	18.8%	16.3%	10.2%	4.1%
Event‐driven oral PrEP	28.0%	23.0%	14.1%	12.8%	18.0%	4.1%
Injectable PrEP	24.6%	19.7%	19.5%	19.0%	10.9%	6.3%
PrEP implant	12.6%	13.6%	17.3%	17.3%	21.9%	17.2%
bnABs	4.5%	11.6%	14.6%	22.6%	24.3%	22.3%
Rectal douche	3.1%	8.5%	15.8%	11.9%	14.6%	46.0%

Abbreviations: bnABs, broadly neutralizing antibodies; PrEP, pre‐exposure prophylaxis.

In the rank order regression model, daily oral PrEP was preferred over all next‐generation modalities (OR vs. daily oral PrEP: event‐driven oral PrEP = 0.89, *p* = 0.058; injectable PrEP = 0.83, *p* = 0.005, PrEP implant = 0.48, *p* < 0.0001; bnAb infusion = 0.38, *p* < 0.0001; PrEP rectal douche = 0.24, *p* < 0.0001) (Table 4).

**Table 4:** Correlates of preferred PrEP modality rankings among young men who have sex with men in the United States

	Event‐driven oral		Injectable		Implant		bnAb		Rectal douche		Omnibus omnibus tests		
	OR	*p*‐value	OR	*p*‐value	OR	*p*‐value	OR	*p*‐value	OR	*p*‐value	Unadj. models	Adj. models	
**Age**											**0.0028**	**0.0278**	^§^
15–17 (ref.)	0.74	0.65	**0.78**	**0.03**	**0.37**	**0.006**	**0.35**	**0.06**	0.21	0.45			
18–21	0.87		**0.64**		**0.35**		**0.30**		0.28				
21–24	0.91		**0.94**		**0.55**		**0.43**		0.23				
**Race**											0.2942		
White (ref.)	0.88	0.90	0.81	0.74	0.45	0.49	0.33	0.05	0.21	0.11			
Non‐White	0.90		0.85		0.49		0.43		0.27				
**Ethnicity**											0.8665		
Latino (ref.)	0.85	0.70	0.85	0.87	0.51	0.53	0.42	0.40	0.24	0.99			
Non‐Latino	0.81		0.87		0.56		0.48		0.24				
**Sexual orientation**											0.6694		
Gay (ref.)	0.85	0.16	0.84	0.93	0.47	0.60	0.37	0.35	0.24	0.35			
Not gay	1.06		0.82		0.51		0.43		0.27				
**Food insecurity**											0.0857	**0.0261**	^§^
Yes (ref.)	**0.68**	**0.0455**	0.78	0.65	0.51	0.62	0.39	0.90	0.19	0.10			
No	**0.94**		0.85		0.47		0.38		0.26				
**PrEP use**											**<0.0001**	**0.0001**	^§^
Never (ref.)	**1.04**	**<0.0001**	0.80	0.56	**0.42**	**0.005**	**0.37**	**0.02**	**0.26**	**0.04**			
Current use	**0.50**		0.81		**0.51**		**0.29**		**0.16**				
Past use	**0.88**		1.02		**0.87**		**0.60**		**0.25**				
**Ever HIV test**											**<0.0001**	**0.0297**	^§^
Yes (ref.)	0.88	0.7	**0.94**	**<0.001**	**0.53**	**<0.001**	**0.42**	**0.002**	0.24	0.64			
No	0.93		**0.54**		**0.31**		**0.26**		0.23				
**Ever STI diagnosis**											**0.0002**		^♯^
Yes (ref.)	0.78	0.24	**1.05**	**0.03**	**0.68**	**0.001**	0.44	0.17	0.23	0.67			
No	0.93		**0.76**		**0.42**		0.36		0.24				
**Condomless anal sex**											**0.0014**	**0.0134**	^§^
Any (ref.)	**0.72**	**0.002**	**0.69**	**0.02**	0.49	0.6	0.34	0.22	0.23	0.53			
None	**1.06**		**0.95**		0.46		0.40		0.25				
**Insurance**											**0.0244**	0.3998	^§^
None (ref.)	0.74	0.53	**0.52**	**0.006**	**0.35**	**0.03**	0.33	0.14	0.21	0.81			
Own insurance	0.85		**1.10**		**0.60**		0.46		0.25				
Parents’ insurance	0.93		**0.78**		**0.44**		0.35		0.24				
**Primary care provider**											0.8528		
Yes (ref.)	0.89	0.98	0.85	0.55	0.46	0.50	0.38	0.69	0.24	0.88			
No	0.89		0.76		0.52		0.41		0.25				
**Healthcare needs met**											**0.0418**	**0.0535**	^§^
Yes (ref.)	0.88	0.73	0.87	0.19	0.50	0.15	0.23	0.12	0.39	0.69			
No	0.93		0.70		0.39		0.30		0.37				
**Any drug use**											**0.0206**	0.273	^§^
Yes (ref.)	0.84	0.73	0.97	0.31	**0.77**	**0.003**	0.49	0.11	0.28	0.33			
No	0.90		0.80		**0.44**		0.36		0.23				
**Alcohol use**											**0.0371**		
2+ drinks/week (ref.)	0.45	0.13	0.22	0.55	**0.62**	**0.02**	**1.03**	**0.05**	0.92	0.74			^♯^
<2 drinks/week	0.36		0.25		**0.43**		**0.77**		0.88				

Notes: Odds ratios (ORs) in the table refer to the odds of selecting each of the modalities in comparison to daily oral PrEP (our reference category).

*p*‐values in the modality columns refer to the hypothesis of whether ORs for modalities are equal across all categories of potential correlates we examined.

Omnibus tests (last two columns) examined the significance of each potential correlate across all modalities in unadjusted and adjusted models. *p*‐values in these columns examine whether ORs were different between categories of potential correlates across all modalities.

Bolded values indicate results with *p*‐value < 0.05.

Abbreviations: bnABs, broadly neutralizing antibodies; HIV, human immunodeficiency virus; PrEP, pre‐exposure prophylaxis; STI, sexually transmitted infections.

^§^ Included in adjusted regression models (Omnibus *p*‐value < 0.10 in unadjusted models, except STI diagnosis and alcohol use).

^♯^ Not included in adjusted models due to high collinearity with another variable.

### 3.3 Correlates of preferred products

While the magnitude differed across levels of the potential correlates examined (described below), the odds of selecting daily oral PrEP was not significantly lower than the odds of choosing any next‐generation modality. Notably, over 90% of individuals ranking *daily oral PrEP* first agreed or strongly agreed that they chose this product due to ease of use, perceived protection against HIV and lack of discomfort (Table 5).

**Table 5:** Reasons for top‐ranked PrEP product among young men who have sex with men in the United States

	Agree/strongly agree with reasons for selecting top choice *N* (%)					
	Daily oral PrEP *n* = 191	Intermittent oral PrEP *n* = 197	Rectal douche *n* = 22	Injectable PrEP *n* = 173	PrEP implant *n* = 89	bnAbs *n* = 32
Would be easy to use	189 (99.0%)	194 (98.5%)	19 (86.4%)	160 (92.5%)	85 (95.5%)	29 (90.6%)
Would have less concerns about side effects	166 (86.9%)	160 (81.2%)	16 (72.7%)	117 (67.6%)	57 (64%)	22 (68.8%)
Would NOT impact daily routine	170 (89.0%)	189 (95.9%)	17 (77.3%)	160 (92.5%)	86 (96.6%)	28 (87.5%)
Would NOT be uncomfortable to use	177 (92.7%)	184 (93.4%)	17 (77.3%)	132 (93.1%)	78 (87.6%)	22 (68.8%)
Would NOT worry about remembering to take	107 (56.0%)	135 (68.5%)	18 (81.8%)	161 (93.1%)	88 (98.9%)	30 (93.8%)
Would NOT worry about having product stolen	152 (79.6%)	153 (77.7%)	17 (77.3%)	162 (93.6%)	86 (96.6%)	27 (84.4%)
Would NOT worry about people finding out I'm taking	134 (70.2%)	142 (72.1%)	17 (77.3%)	156 (90.2%)	85 (95.5%)	29 (90.6%)
Would NOT worry about my use of drugs and alcohol interfering with PrEP	106 (55.5%)	105 (53.3%)	16 (72.7%)	127 (73.4%)	53 (59.6%)	23 (71.9%)
Would make sex life more pleasurable	162 (84.8%)	160 (81.2%)	19 (86.4%)	156 (90.2%)	74 (83.1%)	21 (65.6%)
Would be able to stop taking when I wanted	171 (89.5%)	191 (97.0%)	20 (90.9%)	108 (62.4%)	27 (30.3%)	19 (59.4%)
Would make me feel more protected against HIV	187 (97.9%)	184 (93.4%)	18 (81.8%)	165 (95.4%)	89 (100%)	29 (90.6%)

Abbreviations: bnABs, broadly neutralizing antibodies; HIV, human immunodeficiency virus; PrEP, pre‐exposure prophylaxis.

#### 3.3.1 Event‐driven oral

Participants who reported any food insecurity in the past 3 months were 28% less likely to prefer event‐driven versus daily oral PrEP than those who did not experience food insecurity (*p* < 0.05). Moreover, participants reporting CAS in the past 6 months were 33% less likely to prefer event‐driven to daily oral PrEP (*p* = 0.002). Finally, compared to individuals who had never used PrEP, current users were 52% less likely to prefer event‐driven over daily oral PrEP (*p* < 0.0001) (Table 4).

The highest endorsed reasons for ranking *event‐driven oral PrEP* first included ease of use, ability to stop taking it, no impact on daily routine, perceived protection against HIV and lack of discomfort (Table 5).

#### 3.3.2 Injectable

Compared to individuals 15–17 and 18–20 years, participants 21–24 years old were 21% and 47% more likely to prefer injectable PrEP to daily oral PrEP (*p* < 0.05). Additionally, participants who had prior HIV tests were 74% more likely to prefer injectables to daily oral compared to those who had not been tested (*p* < 0.001), and participants reporting recent CAS were 27% less likely to prefer injectables to daily oral PrEP (*p* = 0.02).

In bivariate analysis, YMSM with their own health insurance had greater odds of preferring injectables over daily oral PrEP compared to those uninsured or covered by their parents’ plan. However, these differences did not remain significant in adjusted models (*p* = 0.40) (Table 4).

The highest endorsed reasons for ranking *injectable PrEP* first included perceived protection against HIV, not worrying about a stolen product, not worrying about remembering to take it, ease of use, no impact on daily routine, not worrying about people finding out they are taking PrEP, improved sex life and lack of discomfort (Table 5).

#### 3.3.3 Implant

Compared to individuals 15–17 and 18–20 years, participants 21–24 years old were 51% and 57% more likely to prefer implants to daily oral PrEP (*p* < 0.01). Participants reporting prior HIV tests were 74% more likely to prefer implants compared to those without prior tests (*p* < 0.001), and, compared to PrEP‐naïve individuals, past PrEP users were 21% more likely to choose PrEP implants over daily oral PrEP (*p* = 0.001).

In bivariate models, the odds of choosing PrEP implants over daily oral PrEP were greater among individuals with their own insurance, and among individuals reporting any drug use in the past 30 days. Yet, these associations did not remain significant in adjusted models (*p* = 0.40 and *p* = 0.27, respectively) (Table 4).

Reasons for ranking *PrEP implants* first were perceived protection against HIV, not worried about remembering to take it, no impact on daily routine, not worried about stolen product, ease of use and not worried about people finding out they are taking PrEP (Table 5).

#### 3.3.4 bnAb

Participants 21–24 years old were 22% and 43% more likely to prefer bnAb infusions to daily oral PrEP compared to individuals 15–17 and 18–20 years, respectively (*p* = 0.06). Participants who had prior HIV test were 65% more likely to prefer bnAb to daily oral PrEP in comparison to individuals not previously tested (*p* < 0.01). Past PrEP users were 60% more likely to choose bnAb infusions (*p* = 0.03) over daily oral PrEP compared to individuals who had never used PrEP (Table 4).

For *bnAb infusions*, the highest endorsed reasons included not worried about remembering to take it, ease of use, not worried about people finding out they are taking PrEP and perceived protection against HIV (Table 5).

#### 3.3.5 Rectal douche

Finally, rectal douches were ranked lowest on average. Only PrEP use was a significant correlate of rectal douche preference, whereas current PrEP users were 38% less likely to prefer rectal douches over daily oral PrEP (*p* = 0.01) compared to never users (Table 4).

The only reason that was endorsed by ≥90% of participants who ranked rectal douche as their top choice was the ability to stop taking it (Table 5).

## 4 DISCUSSION

The present study—which is to our knowledge the first to quantitatively explore in substantial depth the acceptability and preferences for a broad range of PrEP products among YMSM—used multiple innovative methods to explore both within and between product preferences.

Using a discrete‐choice conjoint analysis, which simulates actual decision‐making processes for existent and hypothetical PrEP formulations [33], we found that participants prioritized efficacy, absence of side effects and costs across all PrEP products. Additionally, for long‐acting products (e.g. injectable, implants and bnAb), duration of efficacy was also prioritized. Conversely, delivery mechanism and timing of use or delivery did not demonstrate importance. Our findings are aligned with previous studies showing cost, efficacy and side effects to drive preferences for oral and injectable PrEP formulations among adult MSM and other at‐risk U.S. populations [33, 38–40]. These findings should inform the development of these next‐generation products and how interventions and public awareness campaigns introduce these products when they come to market. For example, research on women's contraceptive decision‐making showing efficacy and side effects as key product attributes [41, 42] has informed the development of decision aids to facilitate contraceptive choices [43].

The rank‐choice analysis demonstrated that daily oral PrEP was preferred over all next‐generation modalities, which contradicts prior studies that demonstrated a preference for injectable PrEP among YMSM [24, 44]; however, these prior studies did not provide a broad array of PrEP options and did not take into account the full range of rankings, and as such may have over‐estimated the preference for next‐generation products. Importantly, our findings suggest that the next‐generation products may not be, without additional efforts, a panacea to PrEP expansion. Efforts to address the underlying barriers to PrEP use among YMSM must not assume that simply changing technology will create demand. Rather, an interdisciplinary and multi‐level approach is needed to remove barriers and improve communication about these products [45]. Resources to support these efforts should be prioritized to ensure the optimal uptake of oral PrEP and the newly FDA‐approved bi‐monthly injectable PrEP in the form of cabotegravir extended‐release injectable suspension [3, 46].

Our study also found differences in preference rankings by socio‐demographic and behavioural characteristics. Individuals who were older age, had prior HIV testing history and were insured were more likely to prefer long‐acting PrEP products than their peers, suggesting that individuals with a greater connection to the healthcare system may be more open to long‐acting next‐generation modalities. Conversely, this also suggests that next‐generation PrEP products may not reach, without additional efforts, those with higher HIV risk and larger barriers to PrEP care (e.g. food insecure and not on own insurance). Notably, while preferences for injectable PrEP were not different across PrEP use categories, compared to current PrEP users, individuals who had never used PrEP were significantly more likely to prefer event‐driven oral PrEP and rectal douches. Previous work has shown that event‐driven oral PrEP may appear appealing to some PrEP‐naïve individuals, but challenges related to planning sexual activity may make some of these individuals eventually switch to daily regimens [47]. Additionally, individuals reporting prior PrEP use were significantly more likely to prefer implants, bnAb and rectal douches, which may indicate an interest in novel modalities among individuals who have tried and discontinued daily oral PrEP due to challenges with daily regimens. This may inform how intervention strategies should differ for distinct groups (e.g. early adopters vs. late adopters), and that equity must be centred in all efforts to rollout new products (e.g. ensuring affordable access to next‐generation modalities regardless of health insurance) [48]. Nonetheless, all groups continued to rank daily oral PrEP ahead of next‐generation options, suggesting that in addition to the need for broad outreach as new products are developed and come to market, efforts to increase uptake and adherence to oral PrEP, which has proven efficacy and an established long‐term safety profile, must be continued and strengthened.

These findings should be considered in light of some limitations. First, while this was a large, diverse, national survey, recruitment was primarily conducted online, and as such, the sample may not represent all YMSM in the United States. Further studies on modality preferences are needed with other key populations in the HIV epidemic, such as transgender women and people who inject drugs. Additionally, a number of these products are still in development and precise information on their efficacy, side effects and other characteristics was not available when our study was conducted, and as such were presented as hypothetical. Familiarity with next‐generation products was not measured. Although we used descriptions of the products developed and tested in cognitive interviews with YMSM [34], preferences may differ if or when the products are available. Lastly, the conjoint experiments were designed to examine preferred features and willingness to pay for product features; however, because each participant was randomized to review one product, we could not quantitatively compare feature preferences across products.

## 5 CONCLUSIONS

Both low rates of PrEP use and suboptimal adherence among YMSM [7, 10, 12] suggest that new modalities are needed to ensure those at risk for HIV acquisition have access to this potentially transformative prevention tool. While scientists are actively developing and testing the efficacy of new PrEP formulations [49], it is essential that end‐users’ preferences for and concerns about PrEP products are understood and considered. Ensuring the preferences for features within products and preferences across products are considered will increase the acceptability of these products and the eventual penetration and expansion of PrEP across communities.

## COMPETING INTERESTS

KBB received unrestricted research grants from Merck. KHM received unrestricted research grants from Gilead and Merck; on the Scientific Advisory Board: Gilead, Merck and ViiV. The authors report no other competing interests.

## AUTHORS’ CONTRIBUTIONS

PKV, RD and WL performed the research. KBB, KHM, LH‐W, DTdS and JB designed the research study. KBB, PKV and JB analysed the data. KBB and PKV led the writing of the paper. All authors provided edits to the paper, and have read and approved the final manuscript.

## FUNDING

This work was made possible through support from the National Institute of Child Health and Human Development (NICHD) (U19HD089881).

## DISCLAIMER

The content is solely the responsibility of the authors and does not necessarily represent the official views of the funding agencies.

## Data Availability

The data that support the findings of this study are available from the corresponding author upon reasonable request.
